# Supplementary material for: Verification of Saliva Matrix Metalloproteinase-1 as a Strong Diagnostic Marker of Oral Cavity Cancer
Source: Cancers (Basel). 2020 Aug 13;12(8):2273. doi: 10.3390/cancers12082273 (PMC7463746; doi:10.3390/cancers12082273)
Supplement: Supplementary file 1 [file cancers-12-02273-s001.zip › cancers-857756 Supplementary Material.docx]

Supplementary Material: Verification of Saliva Matrix Metalloproteinase-1 as a Strong Diagnostic Marker of Oral Cavity Cancer

Ya-Ting Chang, Lichieh Julie Chu, Yen-Chun Liu, Chih-Jou Chen, Shu-Fang Wu, Chien-Hua Chen, Ian Yi-Feng Chang, Jun-Sheng Wang, Tzong-Yuan Wu, Srinivas Dash, Wei-Fan Chiang, Sheng-Fu Chiu, Shin-Bin Gou, Chih-Yen Chien, Kai-Ping Chang and Jau-Song Yu

| **A** |  |
| --- | --- |
| **B** |  |

**Figure S1.** Functional evaluation of human MMP-1-specific mouse mAbs. Four clones of MMP-1 mAbs (6-2, 20-4, 31-34, and 73-1) were tested for application in in-house–established ELISAs. Recombinant MMP-1 (50 ng/well) was coated onto microplates and reacted with 0–50 ng/mL MMP-1 mAb or HRP-conjugated Abs by indirect (**A**) or direct (**B**) ELISA. The specificity of MMP-1 mAbs was evaluated by measuring OD450. Data showed that OD450 values increased with increasing concentrations of Abs, indicating specific reactivity between the MMP-1 mouse mAbs and recombinant MMP-1.

| **A** | 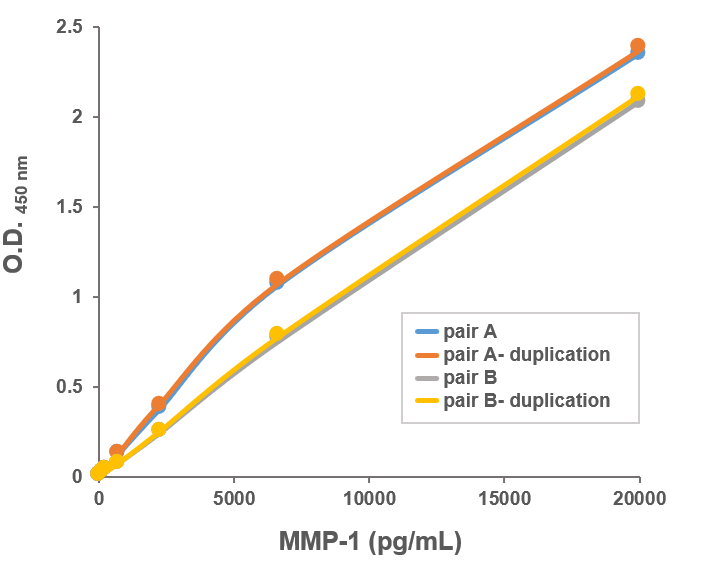 |
| --- | --- |
| **B** | 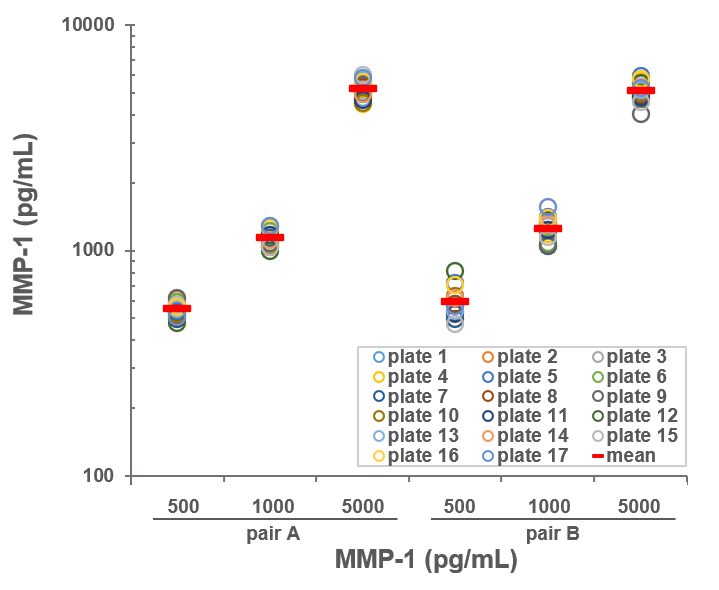 |
| **C** | 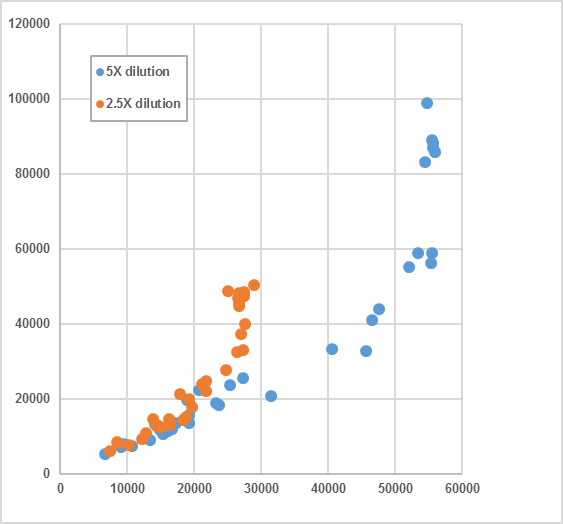 **MMP-1 (pg/mL), pair B-ELISA**  **MMP-1 (pg/mL), pair A-ELISA** |

**Figure S2.** Initial data of pair A- and pair B-ELISA. (**A**) The representative standard curves for determination of recombinant MMP-1 (from 27.4 pg/mL to 20,000 pg/mL) by pair A- and pair B- ELISA in duplication. (**B**) The observed concentration of the three controls (500, 1000 and 5000 pg/mL) determined by pair A- and pair B-ELISA. (**C**) MMP-1 concentrations in the saliva samples from 35 OSCC patients determined by pair A- and pair B-ELISA.

**Table S1** (separate Excel file “Tables S1-S3.xlsx”): Characteristics of study groups and salivary MMP-1 concentration in patients, determined by pair A- and pair B-ELISA.

**Table S2** (separate Excel file “Tables S1-S3.xlsx”): Salivary MMP-1 concentration in patients in the OSCC group, determined by pair A- and pair B-ELISA.

**Table S3** (separate Excel file “Tables S1-S3.xlsx”): Sensitivity, specificity, and accuracy for discriminating OSCC from non-OSCC groups when using salivary MMP-1 at specific cut-off values.
